# Translation and validation of chinese version of MDASI immunotherapy for early-phase trials module: a cross-sectional study

**DOI:** 10.1186/s12912-023-01217-9

**Published:** 2023-05-22

**Authors:** Xiaodan Wu, Jingyue Xie, Xiumei Lin, Limei Hua, Peirong Ding, Shuyue Liu, Simei Shi

**Affiliations:** 1 Sun Yat-sen University Cancer Center, Guangzhou, Guangdong Province China; 2 Unit of Psychiatry, Institute of Translational Medicine, Faculty of Health Sciences, University of Macau, Macao, SAR China; 3 School of Nursing, Sun Yat-sen University, Guangzhou, Guangdong Province China

**Keywords:** MDASI, Validation, Chinese, Immunotherapy, MDASI-Immunotherapy EPT-C

## Abstract

**Background:**

During immunotherapy treatment and survival, identifying symptoms requires a standardized and validated assessment tool. The aim of this study was to translate, validate and use the Chinese version of the Immunotherapy of the M.D. Anderson Symptom Inventory for Early-Phase Trials module (MDASI-Immunotherapy EPT) to assess the symptom burden of cancer patients receiving immunotherapy in China.

**Methods:**

The MDASI-Immunotherapy EPT was translated into Chinese using Brislin’s translation model and the back-translation method. In total, 312 Chinese-speaking colorectal cancer patients receiving immunotherapy were enrolled in the trial from August 2021 to July 2022 after receiving definitive diagnoses in our cancer center. The reliability and validity of the translated version was evaluated.

**Results:**

Cronbach’s α values were 0.964 and 0.935 for the symptom severity and interference scales, respectively. Significant correlations were found between the MDASI-Immunotherapy EPT-C and FACT-G scores (-0.617–0.732, P < 0.001). Known-group validity was supported by significant differences in the scores of the four scales grouped by ECOG PS (all P < 0.01). The overall mean subscale scores for the core and interference subscales were 1.92 ± 1.75 and 1.46 ± 1.87, respectively. Fatigue, numbness/tingling, and disturbed sleep had the highest scores for the most serious symptoms.

**Conclusion:**

The MDASI-Immunotherapy EPT-C showed adequate reliability and validity for measuring symptoms among Chinese-speaking colorectal cancer patients receiving immunotherapy. The tool could be used in clinical practice and clinical trials to gather patients’ health and quality of life data and manage their symptoms in a timely manner in the future.

## Background

Patients with cancer often suffer from many physical and psychological symptoms, including pain, fatigue, and depression. The severity of symptoms profoundly affects their quality of life and functional status [1, 2]. With rapid advances in new therapeutic strategies, such as immunotherapy, the survival of cancer patients has greatly improved [3]. However, multiple immune-related adverse events (irAEs) have emerged due to disruption of immune balance [4], and the incidence of irAEs attributed to single-agent immune checkpoint inhibitor (ICI) therapy can reach as high as 90% [5]. Therefore, patients’ symptoms are further aggravated, indicating the need for effective symptom management [6]. Active management of the symptom burden can reduce or even prevent immunotherapy complications and improve patients’ quality of life while minimizing treatment delays or early discontinuation of treatment plans [7]. The symptoms influence the patient’s activities of daily living (ADL) and may cause life-threatening disorders [8]. An important factor in accurately identifying and assessing symptoms is using a standardized and validated assessment tool. Currently, there is no available tool to assess the symptom burden of immunotherapy treatment adapted for Chinese patients.

In recent years, many scholars have been working on tools for assessing symptoms in cancer patients, which are both universal and specific. Assessment tools play an important role in determining the treatment of cancer patients. The MD Anderson Symptom Inventory (MDASI) is a reliable and valid instrument for measuring common cancer-related symptoms and manifestations [9]. Recently, specific modules for different types of cancer have been developed and psychometrically validated, which can measure the severity and impact of multiple symptoms associated with cancer and its treatment. It can be further developed to assess different cancer types by adding specific items [10–13]. A review evaluating the psychometric properties of 57 symptom instruments found that the MDASI appeared to be the best among all tools for clinical use [14]. A module of the MDASI for measuring immunotherapy symptom burden for early-phase trials in cancer patients (MDASI-Immunotherapy EPT) was recently developed for cancer patients receiving immunotherapy and was found to be a valid, reliable, and concise tool [15]. In this study, we aim to examine and validate the structure of the Chinese version of the MDASI-Immunotherapy EPT (MDASI-Immunotherapy EPT-C) and culturally adapt it in the context of contemporary Chinese.

## Methods

### Study design

This was a cross-sectional study of patients with colorectal cancer. The Ethics Committee of Sun Yat-sen University Cancer Center approved the study (approval no. B2021-361-01). All participants provided written informed consent.

Participants.

The MDASI-Immunotherapy EPT-C was administered to a cross-sectional sample of 312 colorectal cancer patients treated at Sun Yat-sen University Cancer Center from August 2021 to July 2022. Patients were eligible if they (1) had a pathological diagnosis of cancer; (2) were undergoing immunotherapy; (3) were older than 18 years old; (4) were able to communicate orally and in written Chinese; and (5) were willing to sign a written informed consent document. Patients were excluded if they had mental illness or cognitive impairment.

### Measurements

Sociodemographic and disease characteristics were collected by a self-administered questionnaire: age, sex, education, marital status, cancer site, cancer stage, and prior treatment. The patient’s functional ability was assessed using Eastern Cooperative Oncology Group (ECOG) Performance Status (PS) [16]. The scores range from 0 to 4 and were measured by physicians.

The MDASI-Immunotherapy EPT was developed by Mendoza et al. in 2020 ^15^. It contains 26 items grouped into two subscales, which require patients to rate the severity of their symptoms and degree of interference over the past 24 h. The degree of all symptom and interference scores in the MDASI-Immunotherapy EPT-C is expressed as numbers from 0 (“not present” or “does not interfere”) to 10 (“as bad as you can imagine” or “interfered completely”). The original version has an internal consistency (Cronbach’s alpha coefficient) of 0.89–0.95.

The Functional Assessment of Cancer Therapy-General (FACT-G, version 4) is a cross-culturally acceptable cancer-specific health-related quality of life (HRQL) questionnaire that comprises four subscales: Physical Well-being (PWB), Social & Family Well-being (SFWB), Emotional Well-being (EWB), and Functional Well-being (FWB) [17]. These are rated on a 5-point scale ranging from 0 (not at all) to 4 (very much). Scores from the Physical and Emotional Well-being scales (with the exception of one item) are reversed. A total score is derived by summing the scale scores from all four subscales (range 0-108). Higher subscale scores represent better health, functioning, or well-being. The Chinese version has been validated by Yu et al. and has an internal consistency reliability of 0.85 [18].

### Scale translation and linguistic validation

The MDASI-Immunotherapy EPT comprises 19 MDASI items (13 core symptom items plus 6 interference items) and seven immunotherapy-specific items [15]. This instrument showed good reliability and validity. The MDASI-C translated by Wang XS in 2004 was validated in 249 cancer patients and used directly [19].

The MDASI-Immunotherapy EPT was translated using Brislin’s model of forward and backward translation [20]. After obtaining permission from the MDACC Symptom Research Group, two Chinese researchers fluent in English separately translated the seven immunotherapy symptom items into Chinese characters. Then, two other bilingual translators who had not seen the original English items back-translated the Chinese translation into English. This translation/back-translation procedure was repeated until the Chinese version adequately matched the original version. Subsequently the original version, Chinese version and retranslation into the original language were evaluated by an expert panel consisting of eight academic and clinical experts. These experts rated the cultural relevance and consistency of each item by a 4-point scale ranging from 1 (inappropriate) to 4 (very appropriate). The Chinese version would be revised if the expert panel give suggestions.

The Chinese version of the immunotherapy symptom items was tested on 20 randomly selected patients to determine whether the instructions, items, and options were clear and easily understandable and whether they felt confused or offended by the items.

### Statistical analysis

Reliability was assessed based on internal consistency. The internal consistency was evaluated by calculating Cronbach’s α coefficient, which ranges from 0 to 1. A Cronbach’s alpha value of 0.70 or higher indicates good internal consistency [21]. Validity was assessed by content validity, construct validity, known-group validity and criterion validity. Content validity was calculated for each item and subscale by evaluating the items using the content validity index (CVI). The significance of the content validity index above 0.80 indicates an acceptable validity. Construct validity was established using principal axis factor analysis with direct oblimin rotation. The Kaiser‒Meyer‒Olkin (KMO) test confirmed sample adequacy, and a KMO value > 0.5 indicates an acceptable structural validity. Criterion validity was tested by the correlation coefficient, and FACT-G was used as an external criterion. In addition, known-group validity comparisons were examined by comparing the scores between patients’ physical status. All statistical analyses were performed by SPSS (V.26; IBM, Armonk, New York, USA), and statistical significance was set at 0.05.

## Results

### Patient characteristics

The mean ± standard deviation age was 47.03 ± 13.05 years. The majority of patients were male (65.7%). Patients with an education level more than a college degree accounted for 43.3%. The proportions of patients with stage III and IV cancer were 50.3% and 26.0%, respectively (Table 1).

**Table 1:** Patient demographic and clinical characteristics (*N* = 312)

Patient characteristics	*n* (%)
**Age**	
Mean ± SD (yrs)	47.03 ± 13.05
**Gender**	
Male	205 (65.7)
Female	107 (34.3)
**Education**	
Middle school and below	117 (37.5)
High school	60 (19.2)
College and above	135 (43.3)
**Marital status**	
Married	249 (79.8)
Single (including divorced, widowed)	63 (20.2)
**Stage**	
I	0
II	74 (23.7)
III	157 (50.3)
IV	81 (26.0)
**Prior treatment**	
Immunotherapy	126 (40.4)
Immunotherapy and other treatment	186 (59.6)
**ECOG PS**	
0	225 (72.1)
1	72 (23.1)
2	15 (4.8)

ECOG PS = Eastern Cooperative Oncology Group performance status

### Content validity and cognitive debriefing

The Scale Content Validity Index (S-CVI/Ave) was 0.98 for the overall scale, while the Item-Content Validity Index (I-CVI) ranged between 0.88 and 1.00. It confirmed good content validity of the scale. The Chinese version of the instrument was administered to 20 patients and was evaluated according to ease or difficulty in understanding and answering, using a scale of 1–5, from very difficult to very easy. All of the participants found the scale easy to understand and answer.

### Internal consistency

The MDASI-Immunotherapy EPT subscales showed good internal consistency and reliability. The data showed Cronbach’s alpha coefficient for all symptoms, with the core cancer items (13) measuring 0.952, the immunotherapy symptoms items (13) at 0.920, and the interference items (6) at 0.935 (Table 2). These values are well above the usual minimum criterion for reliability of 0.70.

**Table 2:** Internal Consistency Reliability of MDAIS-Immunotherapy EPT-Chinese

Symptom	Cronbach’s α	Total Cronbach’s α if Item Deleted	
Severity (20)	0.957		
Core items (13)	0.952		
Pain		0.965	
Fatigue		0.964	
Nausea		0.964	
Disturbed sleep		0.964	
Distress/feeling upset		0.964	
Shortness of breath		0.965	
Difficulty remembering		0.965	
Lack of appetite		0.964	
Drowsiness		0.964	
Dry mouth		0.965	
Sadness		0.964	
Vomiting		0.965	
Numbness/tingling		0.965	
Immunotherapy-specific items (13)	0.855		
Rash		0.967	
Diarrhea		0.966	
Pain in the abdomen		0.965	
Swelling of hands, legs, or feet		0.965	
Headache		0.965	
Night sweats		0.965	
Fever and/or chills		0.965	
Interference items (6)	0.935		
Activity		0.965	
Mood		0.965	
Work		0.965	
Relations with others		0.965	
Walking		0.965	
Enjoyment of life		0.965	

### Construct validity

Construct validity was assessed by principal axis factor analysis. The KMO score was 0.947, and Bartlett’s test for sphericity was significant (P < 0.001), indicating that the data were suitable for factor analysis. The MDASI-Immunotherapy EPT-C symptom items (26) generated three possible factors, and the total variance explained by all factors was 68.87%. The factor loadings ranged from 0.521 to 0.845 (Table 3). Factor 1 contained all constitutional symptoms; Factor 2 represented gastrointestinal symptoms; and Factor 3 was related to other immunotherapy-specific symptoms.

**Table 3:** Construct Validity of the M. D. Anderson Symptom Inventory: Baseline Factor Loadings of the Symptom Items (*N* = 312)

Symptom	Factor 1	Factor 2	Factor 3
Distress/feeling upset	**0.796**	0.248	0.232
Fatigue	**0.782**	0.387	0.150
Pain	**0.773**	0.263	0.120
Sadness	**0.742**	0.248	0.353
Difficulty remembering	**0.721**	0.262	0.268
Disturbed sleep	**0.698**	0.340	0.277
Dry mouth	**0.669**	0.342	0.288
Shortness of breath	**0.669**	0.246	0.336
Numbness/tingling	**0.645**	0.444	0.244
Drowsiness	**0.626**	0.434	0.297
Vomiting	0.299	**0.845**	0.094
Nausea	0.362	**0.834**	0.168
Lack of appetite	0.452	**0.752**	0.130
Pain in the abdomen	0.289	**0.683**	0.283
Diarrhea	0.143	**0.672**	0.284
Rash	0.160	0.032	**0.790**
Swelling of hands, legs, or feet	0.273	0.309	**0.697**
Fever and/ or chills	0.371	0.335	**0.623**
Headache	0.397	0.350	**0.616**
Night sweats	0.364	0.477	**0.521**

KMO 0.947

Bold values in each column indicate that they belong to the same factor

### Known-group validity

Known-group validity comparisons were made for the MDASI-Immunotherapy EPT Chinese subscales relative to ECOG PS functional classification. The MDASI-Immunotherapy EPT Chinese differentiated between patients with good versus poor functional status. Patients with an ECOG PS of 0 had lower scores on all items than did patients with an ECOG PS of at least 1 (Table 4).

**Table 4:** Known-Group Validity of MDASI-Immunotherapy EPT by ECOG PS

MDASI-Immunotherapy Subscale	Mean ± SD	*P*
Mean severity (20)		
Good	1.27 ± 1.26	< 0.001
Poor	3.92 ± 1.76	
Mean core symptoms (13)		
Good	1.02 ± 0.99	< 0.001
Poor	3.32 ± 1.71	
Mean specific symptoms (7)		
Good	1.02 ± 1.07	< 0.001
Poor	3.43 ± 1.77	
Mean interference (6)		
Good	0.88 ± 1.36	< 0.001
Poor	2.96 ± 2.15	

### Correlation coefficients

The correlation coefficient was shown by the aggregation validity test between the MDASI-Immunotherapy EPT and the FACT-G. Since age was set as a controlled variable in partial correlation analysis, significant correlations were found for the symptom severity scale vs. the FACT-G physical well-being scale (r = -0.729, P < 0.001), the MDASI-core scale vs. the FACT-G physical well-being scale (r = -0.732, P < 0.001), the immunotherapy-specific scale vs. the FACT-G physical well-being scale (r = -0.617, P < 0.001), and the interference scale vs. the FACT-G physical well-being scale (r = − 0.647, P < 0.001).

### Symptoms severity and inter-item distances

**Fig. 1:**
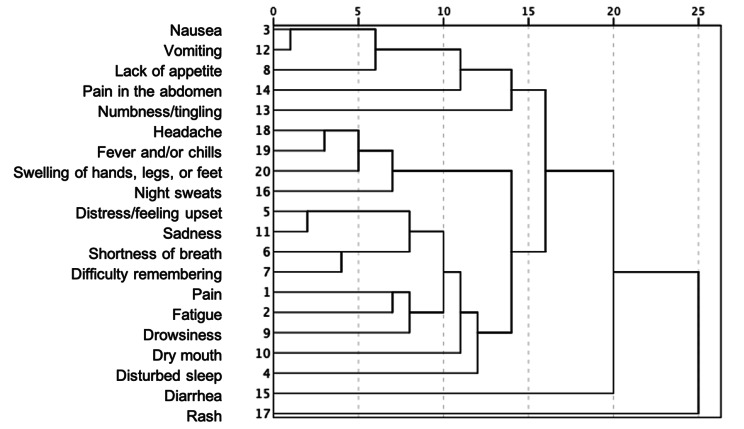
Hierarchical clustering analysis of core and Immunotherapy module items

All symptoms on the MDASI-Immunotherapy EPT-C scale with 0–10 classification were classified into mild (1–4), moderate (5–6), and severe (7–10) based on the score. The overall mean scores for all symptom items (26) and interference items (6) were 1.92 ± 1.75 and 1.46 ± 1.87, respectively. The three most severe symptoms reported were “fatigue” (2.74 ± 2.67), “numbness/tingling” (2.66 ± 2.95), and “disturbed sleep” (2.54 ± 2.80). The mildest symptoms reported were pain in the chest (1.05 ± 1.89) and swelling of the hands, legs, or feet (1.10 ± 1.87). Among the three most serious symptoms, the proportion of patients with severe scores (7–10) accounted for 9.6% for fatigue, 17.0% for numbness/tingling, and 12.8% for disturbed sleep. The three most severe interference items were “work” (1.80 ± 2.36), “mood” (1.70 ± 2.22), and “enjoyment” (1.63 ± 2.22), among which the severe scores (7–10) were 4.5% for work, 4.2% for mood, and 2.9% for enjoyment (Table 5). The correlation between symptoms was explored using cluster analysis, and the relative distance between symptom groups is shown in Fig. 1. Symptoms that were formerly related (left side of the figure) were more relevant than the symptoms that were connected later (right side of the figure).

**Table 5:** Descriptive statistics for the severity of the symptom items of the MDAIS-Immunotherapy EPT, 0–10 scale, in rank order (*N* = 312)

Symptom	Mean Score	SD	% of patients rating item as:			
			0	1–4 (mild)	5–6 (moderate)	7–10 (severe)
Severity						
Fatigue	2.74	2.67	31.7	40.1	18.6	9.6
Numbness/tingling	2.66	2.95	36.9	36.2	9.9	17.0
Disturbed sleep	2.54	2.80	37.5	36.2	13.5	12.8
Lack of appetite	2.43	2.69	36.9	39.4	14.1	9.6
Dry mouth	2.40	2.72	39.7	37.9	12.1	10.3
Diarrhea	2.32	2.72	41.3	33.7	14.1	10.9
Rash	2.31	2.68	38.8	42.3	9.3	9.6
Distress/feeling upset	2.19	2.51	38.8	39.7	15.7	5.8
Sadness	2.10	2.51	41.3	39.1	11.9	7.7
Drowsiness	2.08	2.32	36.9	44.8	13.2	5.1
Difficulty remembering	1.88	2.25	40.1	42.0	13.4	4.5
Nausea	1.86	2.69	52.9	27.9	8.6	10.6
Pain	1.84	2.44	48.7	38.8	6.4	6.1
Shortness of breath	1.79	2.17	43.3	45.2	7.7	3.8
Vomiting	1.79	2.79	56.7	26.3	5.1	11.9
Pain in the abdomen	1.79	2.40	45.8	40.7	5.8	7.7
Night sweats	1.60	2.22	52.2	33.1	10.2	4.5
Headache	1.48	2.11	53.5	35.9	6.4	4.2
Fever and/ or chills	1.24	2.06	61.2	28.2	6.4	4.2
Swelling of hands, legs, or feet	1.10	1.87	64.7	27.6	4.5	3.2
Interference						
Work	1.80	2.36	47.4	35.6	12.5	4.5
Mood	1.70	2.22	46.2	41.3	8.3	4.2
Enjoyment	1.63	2.22	51.9	34.3	10.9	2.9
Walking	1.35	2.21	61.9	25.9	8.0	4.2
Relations	1.33	2.03	55.8	35.2	6.1	2.9
Activity	0.94	1.81	69.6	25.9	2.3	2.2

## Discussion

In this study, the MDASI-Immunotherapy EPT-C was translated and validated. The results endorsed good internal consistency reliability and validity in assessing the symptom burden of receiving immunotherapy.

The items for both symptoms and interference indicated high internal consistency reliability with a Cronbach’s alpha coefficient exceeding 0.90. In the principal axis factor analysis, we obtained three underlying constructs: constitutional (ten items), gastrointestinal (five items), and immunotherapy-specific (five items). These components typified clinically meaningful entities that supplemented each other. The KMO value in this study was 0.947, which was much higher than 0.5, indicating that the scale had good construct validity. Our study showed that the scale was greatly correlated with the FACT-G and supported the measurement of its associated specific symptoms. The FACT-G is the most widely used HRQL assessment in cancer patients [22]. Previous studies have shown that the symptom measurement was more informative in measuring and monitoring specific symptoms [23, 24].

The average score obtained from the core symptoms was different from the distribution reported by Tito Mendoza et al. [15]. Mendoza et al. described pain, fatigue, and disturbed sleep as the three most severe symptoms in general, while in our group, fatigue was the most serious core symptom, followed by numbness/tingling and disturbed sleep. This result may be related to the proportion of cancer types. In their initial study, the most common cancer diagnosis was colorectal cancer (9%), while there were other cancer types. Our study mainly focused on colorectal cancer patients. To assess known-group validity, we applied the same assumption as in the validation study of MDASI-Immunotherapy EPT. The severity of symptom burden and interference of daily activities was correlated with ECOG PS, indicating the sensitivity of MDASI-Immunotherapy EPT-C to predict patient physical condition. The results showed that immunotherapy had little effect on the patients’ activities, relations, and mobility. However, the impact on work and mood was more persistent. These immunotherapy-specific symptoms might continue to affect the daily lives of patients and their families. Thus, health professionals should pay close attention and offer support to these individuals [25].

The validation of this instrument has several clinical and research uses. First, it offers an objective method to evaluate symptoms in Chinese-speaking patients receiving immunotherapy. In clinical trials, an increasing number of clinicians and patients found that understanding patients’ experiences with the effects of new therapies added critical information needed to evaluate these therapies [26]. While patient-reported outcomes were seen as an opportunity for patients to describe their feelings and experiences, proper measurement is needed [27]. Currently, there is a lack of special instruments to measure symptomatic adverse events experienced by patients receiving immunotherapy. The MDASI-Immunotherapy EPT-C could fill this gap. Second, it provides a more reliable tool and can be used to monitor the deterioration of patients’ status during treatment to adjust or redesign therapeutic strategies to mitigate their symptoms, and prevent subjective assessments between physicians.

### Limitations

There were a few limitations to the present study. First, the participants were recruited by convenience sampling. Second, this study was conducted at a single cancer center. These two limitations results may jeopardize the generalizability of our results. However, our study had the strength of a fairly large sample of patients receiving immunotherapy alone or in combination with other cancer therapies. Second, this study only included patients with colorectal tumors. Thus, the MDASI-Immunotherapy EPT-C should be validated in patients with other cancers in the future. Finally, some patients reported that they were experiencing symptoms such as cough and palpitations, while there were no corresponding items. Researchers could consider modifying the scale based on exploratory qualitative research.

## Conclusion

Fatigue, numbness/tingling, and disturbed sleep were the most important symptoms among colorectal cancer patients receiving immunotherapy. The MDASI-Immunotherapy EPT-C demonstrated strong psychometric evidence in Chinese-speaking cancer patients receiving immunotherapy. This instrument could be used in clinical practice to assess patients’ conditions, adjust treatments and manage symptoms in the future.

## Data Availability

The datasets used and analyzed during the current study are available from the author based on reasonable request.

## Abbreviations

MDASI: MD Anderson Symptom Inventory
ECOG PS: Eastern Cooperative Oncology Group Performance Status
ICI: Immune checkpoint inhibitor
FACT-G: The Functional Assessment of Cancer Therapy-General
